# Effects of Urolithin A on Mitochondrial Parameters in a Cellular Model of Early Alzheimer Disease

**DOI:** 10.3390/ijms22158333

**Published:** 2021-08-03

**Authors:** Carsten Esselun, Ellen Theyssen, Gunter P. Eckert

**Affiliations:** Biomedical Research Center, Institute of Nutritional Sciences, Justus-Liebig-University of Giessen, D-35392 Giessen, Germany; carsten.esselun@ernaehrung.uni-giessen.de (C.E.); ellen.theyssen@ernaehrung.uni-giessen.de (E.T.)

**Keywords:** metabolite, polyphenol, mitochondria, neurodegeneration, Alzheimer’s, ellagitannin, urolithin, hormesis

## Abstract

(1) Background: Ellagitannins are natural products occurring in pomegranate and walnuts. They are hydrolyzed in the gut to release ellagic acid, which is further metabolized by the microflora into urolithins, such as urolithin A (UA). Accumulation of damaged mitochondria is a hallmark of aging and age-related neurodegenerative diseases. In this study, we investigated the neuroprotective activity of the metabolite UA against mitochondrial dysfunction in a cellular model of early Alzheimer disease (AD). (2) Methods: In the present study we used SH-SY5Y-APP695 cells and its corresponding controls (SH-SY5Ymock) to assess UA’s effect on mitochondrial function. Using these cells we investigated mitochondrial respiration (OXPHOS), mitochondrial membrane potential (MMP), adenosine triphosphate (ATP) production, autophagy and levels of reactive oxygen species (ROS) in cells treated with UA. Furthermore, we assessed UA’s effect on the expression of genes related to mitochondrial bioenergetics, mitochondrial biogenesis, and autophagy via quantitative real-time PCR (qRT-PCR). (3) Results: Treatment of SH-SY5Y-APP695 cells suggests changes to autophagy corresponding with qRT-PCR results. However, LC3B-I, LC3B-II, and p62 levels were unchanged. UA (10 µM) reduced MMP, and ATP-levels. Treatment of cells with UA (1 µM) for 24 h did not affect ROS production or levels of Aβ, but significantly increased expression of genes for mitochondrial biogenesis and OXPHOS. Mitochondrial Transcription Factor A (TFAM) expression was specifically increased in SH-SY5Y-APP695. Both cell lines showed unaltered levels of peroxisome proliferator-activated receptor gamma coactivator 1-alpha (PGC1α), which is commonly associated with mitochondrial biogenesis. Results imply that biogenesis might be facilitated by estrogen-related receptor (ESRR) genes. (4) Conclusion: Urolithin A shows no effect on autophagy in SH-SY5Y-APP695 cells and its effect on mitochondrial function is limited. Instead, data suggests that UA treatment induces hormetic effects as it induces transcription of several genes related to mitochondrial biogenesis.

## 1. Introduction

Mitochondrial dysfunction (MD) is a hallmark of aging and many age-related diseases [1,2] including Alzheimer’s disease (AD) [3,4,5]. Mitochondrial dysfunction is characterized by changes of mitochondrial bioenergetics [6,7,8], e.g., impairment of the oxidative phosphorylation (OXPHOS) leading to lower mitochondrial membrane potential (MMP) and adenosine triphosphate (ATP) production which also corresponds with an excessive production of reactive oxygen species (ROS) [9], but also alterations to mitochondrial dynamics like fusion and fission [10,11]. One functionality of mitochondrial fusion and fission [12,13] is the selective separation of dysfunctional compartments of mitochondria for later removal via autophagy or more specifically mitophagy [10,14]. This process targets mitochondria to autophagosomes, which are fused to lysosomes for degradation and therefore represents an important mechanism for mitochondrial turnover. Formation of autophagosomes is accompanied by the conversion of microtubule-associated proteins 1A/1B light chain 3B (LC3B-I) to its phosphatidylethanolamine-conjugated form (LC3B-II) and its recruitment [15]. Microtubule-associated protein 1A/1B-light chain 3 (MAP1LC3), represents a widely used marker that first was identified to associate with autophagosomal structures. In AD, evidence suggests that autophagic processes are impaired [16] and since mitochondria lack other mechanisms to repair MD-related changes, the increasing MD is an unavoidable consequence of the aging process [1,17]. Once MD reaches a certain threshold, pathophysiological processes leading to neurodegenerative disease might be initiated [18,19]. Two common pathologies associated with AD are changes to amyloid precursor protein (APP) procession leading to accumulation of amyloid beta peptide (Aβ) [20] or the hyperphosphorylation of tau protein [21]. In return, both of these pathological processes are also affecting mitochondrial bioenergetics [22,23,24,25,26].

**Figure 1:**
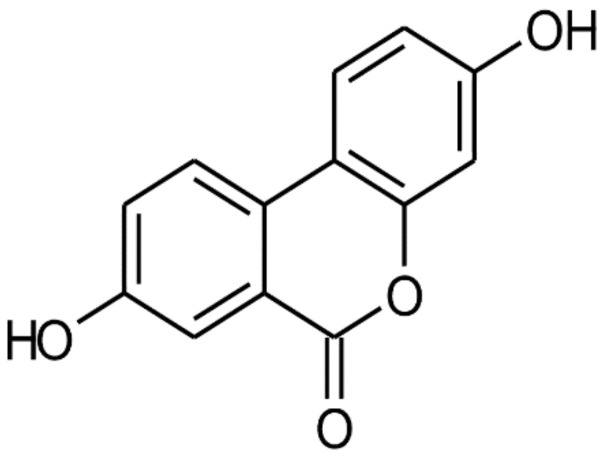
Chemical structure of compound Urolithin A.

Urolithin A (UA) (see Figure 1), generated by gut bacteria from their metabolic precursors, the ellagitannins, which are found in the pomegranate fruit and walnuts [27], has been shown to cross the blood-brain barrier in-silico [28], as well as in-vitro [29]. This metabolite was found to be a potent inducer of mitophagy and improve mitochondrial function in the model organism *Caenorhabditis elegans* [27]. Other studies in sw620 CRC cells [30] or MIN6β [31] cells found generally increased autophagy induced by UA, and Ahsan et al., specifically found autophagy increased while mitophagy was unaltered [32]. Low doses of UA have also been reported to provide neuroprotection against H_2_O_2_ induced oxidative stress in N2a [33] and SH-SY5Y [34,35] cells. Furthermore, multiple studies also reported increased mitochondrial function and enhanced mitochondrial biogenesis facilitated by UA [27,36,37,38,39].

In order to evaluate UA’s effect on mitochondrial dysfunction, aging and AD, we aimed to investigated several markers of mitochondrial function, e.g., respiration of cells, MMP and ATP levels, ROS production, autophagy, and a variety of genetic markers to identify possible molecular mechanisms induced by UA in neuronal cells. For this purpose, we used SH-SY5Y neuroblastoma cells, transfected with the amyloid-precursor protein 695 (SY5Y-APP695) as an established model for early AD [22,40,41]. Cells transfected with an empty pCEP4 vector were used as controls (SY5Ymock).

## 2. Results

### 2.1. Urolithin A Does Not Affect Mitochondrial Function in SY5Y-APP695 Cells

**Figure 2:**
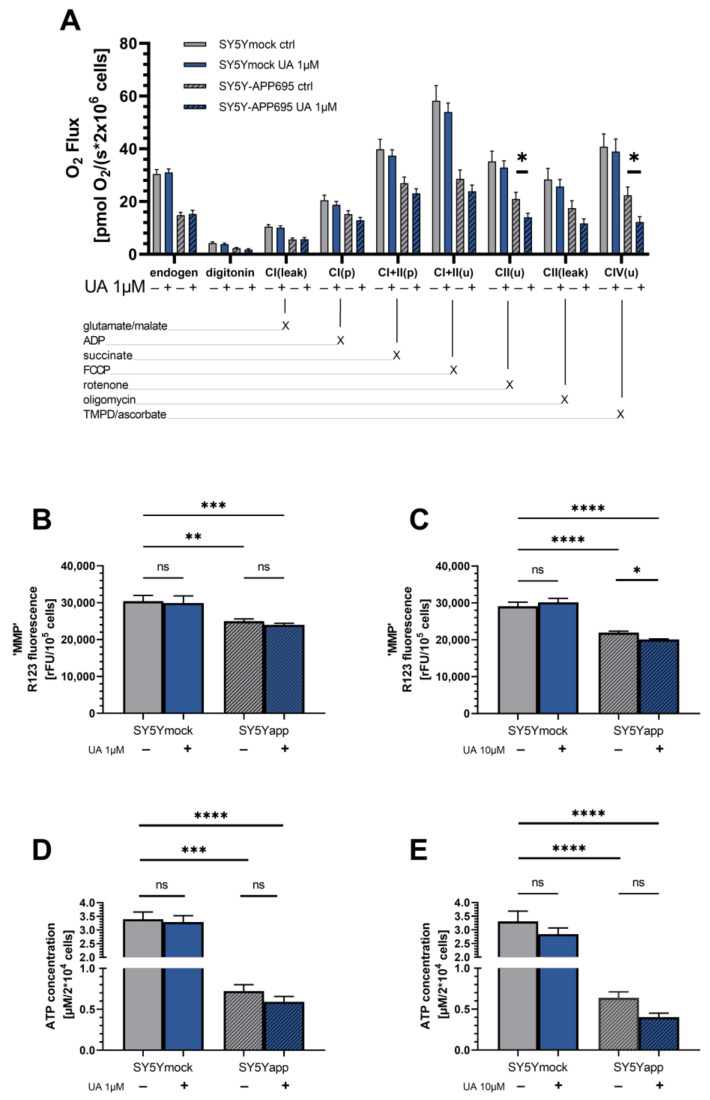
(**A**): Oxygen consumption of 20^6^ SY5Ymock cells and SY5Y-APP695 cells treated with ctrl or 1 µM UA. N = 9–15. Activity of OXPHOS complexes were assessed via addition of several substrates, inhibitors or uncouplers. Which substance was added in which stage of the experiment is marked with “X”. (**B**,**C**) Mitochondrial membrane potential measured of SY5Ymock cells and SY5Y-APP695 cells treated with 1 µM UA (**B**) or 10 µM UA (**C**). N = 7–11; (**D**,**E**) ATP levels of SY5Ymock and SY5Y-APP695 cells treated with 1 µM UA (**D**) or 10 µM (**E**). N = 11–13 Displayed are means ± SEM. Statistical significance was tested via two-way ANOVA followed by a false discovery rate correction according to Benjamini, Krieger, and Yekutieli. Significance is displayed as: ^ns^ *p* > 0.05, * *p* < 0.05, ** *p* < 0.01; *** *p* < 0.001, **** *p* < 0.0001. List of all statistical parameters and comparisons can be found in Supplementary Table S1.

We investigated whether UA was modulating mitochondrial function. For this, we assessed OXPHOS respiration, ATP levels and changes of the MMP. Displayed in Figure 2A is the oxygen flux of SY5Ymock and SY5Y-APP695 cells. Comparing both cell lines’ endogenous respiration as an indicator of normal cell respiration, the APP695 transfection clearly reduced the respiration of the cells by around 40% (**** *p* < 0.0001). Further to note, while uncoupled physiological respiration (CI + II_E_) reflects maximal O_2_ consumption compared to all other stages of the experiment in SY5Ymock cells, SY5Y-APP695 cells are already working at maximum capacity in a physiological state (CI + II_P_). This is indicated by little to no changes to the OXPHOS following addition of FCCP uncoupling respiration from the MMP.

Treatment with 1 µM UA did not alter the respiration of SY5Ymock cells (Figure 2A). Both groups were virtually identical across all stages of the experiments. In SY5Y-APP695 cells however, treatment with 1 µM UA showed a trend for lower respiration of cells in most stages of the experiment with the exception of CI_(leak),_ which was virtually identical to ctrl. Uncoupled CII and CIV respiration were significantly reduced by UA treatment (CII_(U)_: * *p* = 0.043; CIV_(U)_: * *p* = 0.042). Since complex activities were affected by UA treatment in SY5Y-APP695 cells, a reduced MMP and ATP production could also be expected. However, examination of MMP and ATP levels revealed that treatment with 1 µM UA did not affect either parameter in the two cell lines (Figure 2B,D) significantly. When cells were treated with a 10-fold increased UA concentration (10 µM) however, MMP was significantly reduced (* *p* = 0.032) and a similar trend was observed for the ATP concentration (Figure 2C,E). Taken together, there have not been any observable effects of UA to attenuate model-specific deficiencies and a 10-fold increased UA concentration might even worsen mitochondrial function in addition to model-specific changes.

Next we investigated citrate synthase (CS) activity, as a marker for mitochondrial mass [42,43]. Since respiration chain complex activities were reduced and mitochondrial function appeared to be unaffected at low concentrations of UA, we investigated whether there were any changes to mitochondrial mass. Generally, there was a strong trend for lower CS activity in SY5Y-APP695 cells compared to SY5Ymock cells (*p* = 0.052). Treatment of SY5Y-APP695 cells with UA did not affect mitochondrial mass in either cell line. 

**Figure 3:**
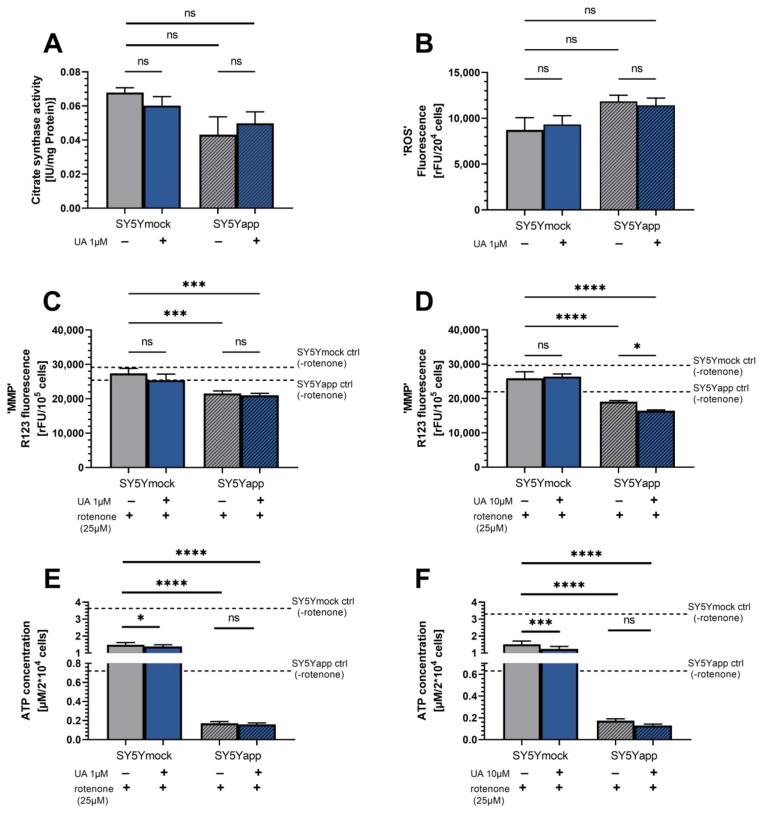
(**A**) Citrate synthase activity of SY5Ymock and SY5Y-APP695 samples. Cells were incubated with either 1 µM UA or its DMSO control (ctrl). Enzyme activity waws adjusted to protein content of the samples. N = 7–13. (**B**) ROS level measured in form of DCFDA/H2DCFDA fluorescence in SY5Ymock and SY5Y-APP695 cells. N = 8; (**C**,**D**) Mitochondrial membrane potential of SY5Ymock and SY5Y-APP695 cells treated with 1 µM UA (**C**) or 10 µM UA (**D**) whose complex I activity was inhibited via addition of 25 µM rotenone. Dashed line corresponds to MMP of uninhibited SY5Ymock/APP695 control cells. N = 7–11; (**E**,**F**) ATP levels of SY5Ymock and SY5Y-APP695 cells treated with 1 µM UA (**E**) or 10 µM (**F**) whose complex I activity was inhibited via addition of 25 µM rotenone. The dashed line shows the basal ATP level of SY5Ymock/-APP695 cells not treated with 20 µM rotenone. Dashed line corresponds to MMP of uninhibited SY5Ymock/APP695 control cells. N = 7–14; Displayed are means ± SEM. Statistical significance was tested via one-way ANOVA followed by a false discovery rate correction according to Benjamini, Krieger and Yekutieli. Significance is displayed as: ^ns^ *p* > 0.05, * *p* < 0.05, *** *p* < 0.001 and **** *p* < 0.0001. List of all statistical parameters and comparisons can be found in Supplementary Table S1.

Impaired OXHOS, especially impaired complex I–III, may induce the generation of reactive oxygen species (ROS). For this reason, we investigated UA’s effects on cellular ROS production (Figure 3B). Generally, SY5Y-APP695 cells tend to show higher ROS compared to SY5Ymock cells, but treatment with 1 µM UA did not improve this. Specific inhibition with complex I-inhibitor rotenone, an established ROS inducer in SY5Y cells [44,45], reduced MMP and ATP production (Figure 3E–H), but was not rescued by UA in either concentration 1 µM or 10 µM. Instead, treatment with 10 µM UA in SY5Y-APP695 cells added to the negative effect of rotenone and reduced MMP further (* *p* = 0.026). In rotenone-treated SY5Ymock cells, 1 µM UA (* *p* < 0.033) and 10 µM (*** *p* = 0.0002) reduced basal ATP levels even further, also adding to rotenone’s effect, while MMP was unaffected in the same groups (Figure 3E,F).

Regarding the results for Aβ-expression, treatment of cells with 1 µM UA had no affect on its production in SY5Y-APP695 cells (Table 1).

Taken together, higher concentrations (10 µM) were required to affect mitochondrial function. Overall, in control cells only minor effects were detectable. Importantly, mitochondrial dysfunction of AD cells appeared tended to be worsened by 10 µM UA on MMP and 1 µM on OXPHOS.

### 2.2. Urolithin A Enhances Gene Expression for OXPHOS and Mitochondrial Biogenesis

**Figure 4:**
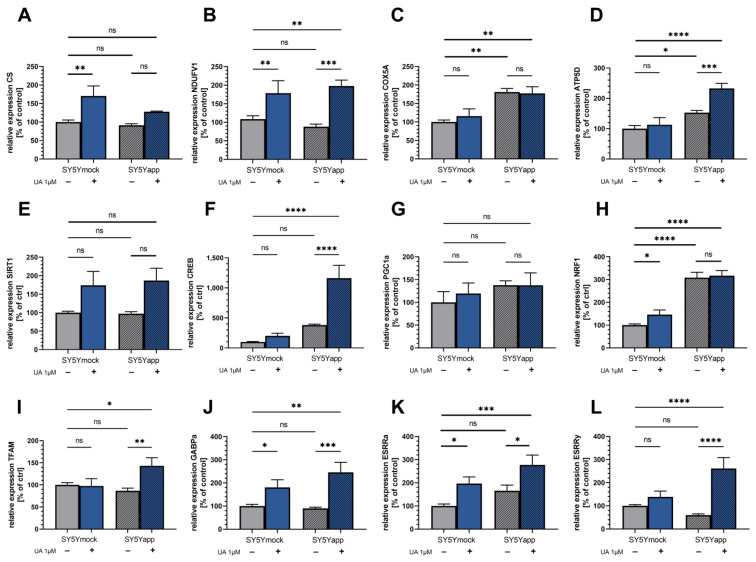
Relative normalized mRNA expression of relevant genes relevant to OXPHOS and mitochondrial biogenesis. Genes related to OXPHOS: (**A**) citrate synthases (CS), (**B**) complex I (NDUFV1), (**C**) complex IV (COX5D), and (**D**) complex V (ATP5D). Genes related to mitochondrial biogenesis: (**E**) Sirtuin 1 (SIRT1), (**F**) cAMP response element-binding protein ranscription factor (CREB), (**G**) Peroxisome proliferator-activated receptor gamma coactivator 1-alpha (PGC1α), (**H**) nuclear respiratory factor 1 (NRF1), (**I**) mitochondrial transcription factor A (TFAM), (**J**) GA Binding Protein Transcription Factor Subunit Alpha (GABPα/NRF2), (**K**) estrogen-related receptor alpha (ESRRα), (**L**) estrogen-related receptor gamma (ESRRγ). Displayed are data of SY5Ymock and SY5Y-APP695 cells which were measured seperately from each other. Data of both cell lines is adjusted to aged SY5Ymock ctrl group = 100%; N = 8–10; Displayed are means ± SEM; Statistical significance was tested via two-way ANOVA followed by a false discovery rate correction (FDRC) according to Benjamini, Krieger, and Yekutieli. Results were normalized to the mRNA expression levels of three housekeeping genes (beta-actine (ACTβ), Glyceraldehyde 3-phosphate dehydrogenase (GAPDH) and Phosphoglycerate Kinase 1 (PGK1)) according to the MIQE guidelines. Significance is displayed as: ^ns^ *p* > 0.05, * *p* < 0.05, ** *p* < 0.01, *** *p* < 0.001 and **** *p* < 0.0001. List of all statistical parameters and comparisons can be found in Supplementary Table S1.

As shown in the Figure 4, gene expression of citrate synthase (CS) and complex I (NDUFV1), were similar in both cell lines’ control cells. Complex IV (COX5A) and complex V (ATP5D) on the other hand showed a higher expression in SY5Y-APP695 compared to SY5Ymock cells (COX5A: ** *p* = 0.001; ATP5D: * *p* = 0.013). In SY5Y-APP695 cells, incubation for 24 h with 1 µM UA significantly increased the gene of ATP5D (*** *p* = 0.0007) and showed a trend to increase CS expression. In both cell lines the same treatment increased NDUFV1 (SY5Ymock, ** *p* = 0.0048; SY5Y-APP695, *** *p* = 0.0009) significantly. COX5A expression showed the greatest difference between both cell lines but was not affected by UA treatment. While not significant in SY5Y-APP695, the increase in CS gene expression was significant in SY5Ymock cells (** *p* = 0.0046). 

**Figure 5:**
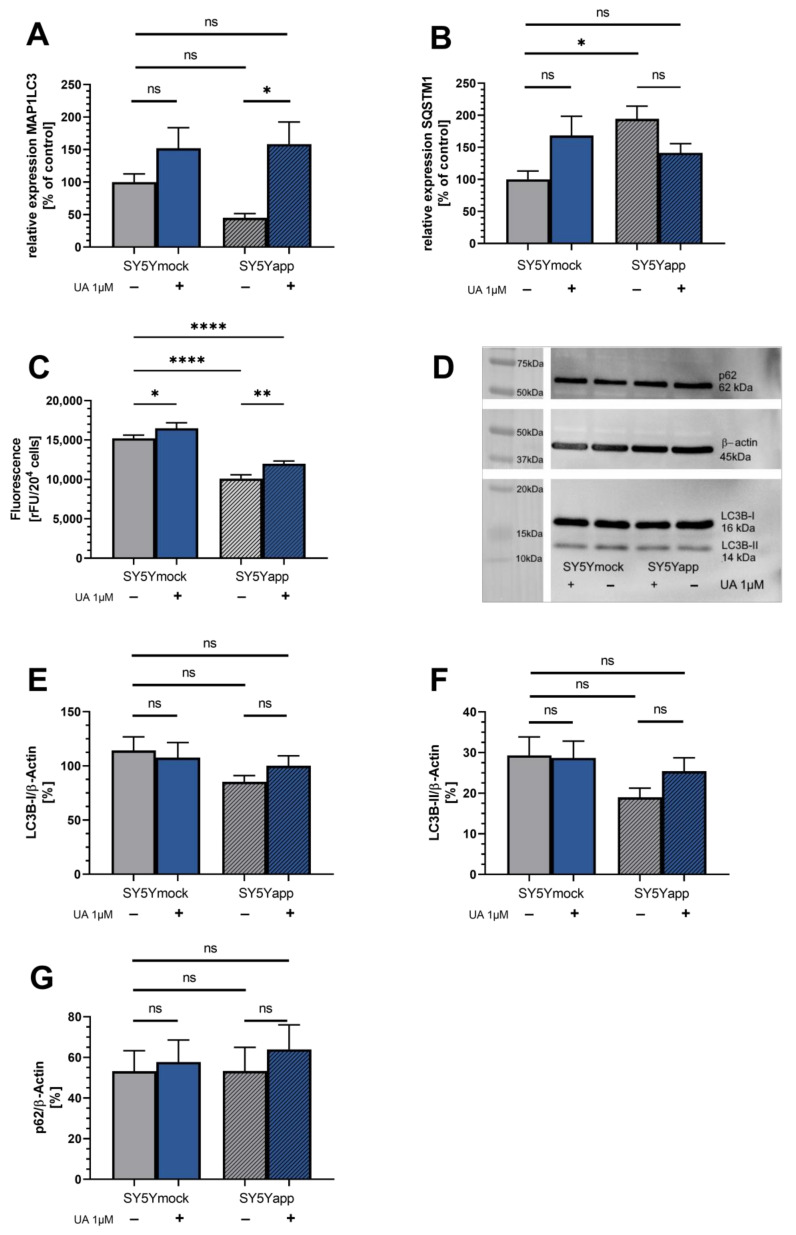
(**A**) Expression of microtubule-associated proteins 1A/1B light chain 3 (MAP1LC3) gene related to autophagy; (**B**) Expression of Sequestosome 1 (SQSTM1) gene related to autophagy. Displayed are data of SY5Ymock (left side) and SY5Y-APP695 cells (right side) which were measured separately from each other. Data of both cell lines is adjusted to SY5Ymock ctrl group = 100%; N = 8–10; Displayed are means ± SEM. Results were normalized to the mRNA expression levels of three housekeeping genes (beta-actine (ACTβ), Glyceraldehyde 3-phosphate dehydrogenase (GAPDH) and Phosphoglycerate Kinase 1 (PGK1)) according to the MIQE guidelines. (**C**) Fluorescence of marker dye binding to autophagosomes of SY5Ymock and SY5Y-APP695 cells. Displayed are means ± SEM. N = 10. (**D**) Western blots of LC3B-I, LC3B-II, and p62 in SY5Ymock and SY5Y-APP695 treated with 1 µM UA or ctrl. β-Actine was used for housekeeping and results have been adjusted to it (Figure 2E–G). Gels were loaded with 15 µg protein. (**E**,**F**) Western blot results from LC3B-I and LC3B-II adjusted to β-actine. Displayed are means ± SEM. N = 9. (**G**) Western blot results from p62 adjusted to β-actine. Displayed are means ± SEM. N = 8. Cells were incubated with either 1 µM UA or its DMSO control (ctrl). Statistical significance was tested via two-way ANOVA followed by a false discovery rate correction according to Benjamini, Krieger, and Yekutieli. Significance is displayed as: ^ns^ *p* > 0.05, * *p* < 0.05, ** *p* < 0.01 and **** *p* < 0.0001. List of all statistical parameters and comparisons can be found in Supplementary Table S1.

PGC1α expression was virtually identical across all groups and cell lines (Figure 5G). SIRT1 and CREB are both inducers of PGC1α gene expression. SIRT did not differ significantly between the cell lines and was not significantly affected by UA treatment. However, strong trends for an increase were observed (SY5Ymock: *p* = 0.071; SY5Y-APP695: *p* = 0.061). CREB was significantly increased by UA treatment in SY5Y-APP695 cells compared to SY5Ymock (**** *p* < 0.0001), but only tended to be increased in the latter after treatment. NRF1, which activates the expression of mitochondrial transcription factor A (TFAM), was increased in SY5Ymock cells after UA treatment (* *p* = 0.042), while being virtually identical in both SY5Y-APP695 groups. Generally, SY5Y-APP695 cells showed a higher expression of this gene (**** *p* < 0.0001). Surprisingly, TFAM which is downstream of NRF1, was significantly increased in SY5Y-APP695 cells (** *p* = 0.0051) but not in SY5Ymock. Since PGC1α appeared to be unchanged, TFAM might be induced via a different signal way. GABPα (subunit of nuclear respiratory factor 2 (NRF2)) was significantly increased in both cell lines after UA treatment (SY5Ymock, * *p* = 0.041; SY5Y-APP695, *** *p* = 0.0008) and similarly estrogen-related receptor alpha (ESRRα) was significantly increased in SY5Ymock cells following UA treatment (* *p* = 0.024) and SY5Y-APP695 cells (* *p* = 0.014). However, ESRRγ was increased in SY5Y-APP695 (**** *p* < 0.0001) cells only, similar to TFAM. 

### 2.3. Urolithin Does Not Affect Autophagy in SY5Y-APP695 Cells

A prominent property of UA is the induction of autophagy; however, up to this date, it is unclear if this effect also occurs in neuronal SY5Ymock and SY5Y-APP695 cells. In general, the marker dye, which binds to autophagosomes in cells, did show a higher fluorescence in mock-cells compared to SY5Y-APP695-cells (**** *p* < 0.0001) suggesting reduced autophagosomes in SY5Y-APP695-cells. Moreover, gene expression of MAP1LC3 tended to be reduced in SY5Y-APP695 cells. Shown in Figure 5A,B is the expression of genes related to the process of autophagy: While MAP1LC3 appears to be reduced in SY5Y-APP695 cells, the opposite is evident for SQSTM1 (* *p* = 0.012). Treatment of SY5Y-APP695 cells with 1 µM UA significantly increased MAP1LC3 (* *p* = 0.010) expression. On the other hand, expression of SQSTM1 tended to be decreased in SY5Y-APP695 cells after UA treatment without reaching significance. However, it still lowered SQSTM1 expression to a level similar to SY5Ymock cells (*p* = 0.21). In SY5Ymock cells, treatment with UA showed a trend to increase expression of SQSTM1 (*p* = 0.06). Looking at the fluorescence of the autophagosome-binding dye revealed that treatment of SY5Ymock cells with 1 µM UA increased the number of autophagosomes in both SY5Ymock (* *p* = 0.015) and SY5Y-APP695 cells (** *p* = 0.0026). This supports the trends indicated by MAP1LC3 gene expression, which tended to be increased in SY5Ymock cells and which was significantly increased in SY5Y-APP695 cells. Looking at results from Western blotting in Figure 5D–G however, we were unable to confirm these results on protein levels. LC3B-I, LC3B-II, and p62 were unaffected by UA treatment in SY5Ymock cells. Untreated SY5Y-APP695 cells tended to have lower LC3B-II levels compared to SY5Ymock cells. UA’s effect on LC3B-II in SY5Y-APP695 was limited and did not significantly improve its content. Protein p62, which is degraded during autophagy, is virtually identical across both cell lines and treatment groups. Taken together, these results suggest that UA might only increase the number of autophagosomes but does not affect their degradation.

## 3. Discussion

Mitochondrial dysfunction plays a prominent role in the aging process and is part of the pathogenesis of neurodegenerative diseases including AD [3,19,46,47]. Mitochondrial dysfunction is characterized by changes of mitochondrial bioenergetics [6,7], excessive production of ROS [9], and altered mitochondrial dynamics [11,48]. Fusion and fission [11,48] are key players in mitophagy [10,14]. Impaired mitophagy leads to the accumulation of damaged mitochondria [10,11] and starts a vicious cycle accelerating cellular loss and subsequently neurodegeneration.

To investigate the effect of UA on mitochondrial function in a cellular model, we looked at both, a control cell line (SY5Ymock) and a model of early Alzheimer Disease, characterized by an APP-transfection, leading to increased production of amyloid beta peptide (SY5Y-APP695), a biochemical phenotype commonly associated with AD [22]. As expected, when comparing both cell lines, severe mitochondrial dysfunction occurred in almost all investigated parameters. Adding to the generally lower respiration in SY5Y-APP695, CI&CII were already working near their maximum capacity in coupled state if compared to uncoupled state. This is in contrast to SY5Ymock cells whose CI&CII respiration still had the capacity to increase after uncoupling with FCCP. These findings are generally in line with the expected effects of increased Aβ peptide production [22,41].

When we looked at general parameters of mitochondrial function, complex activities of OXPHOS mostly tended to be decreased following UA treatment while parameters like MMP or ATP were unaffected or showed trends for a decrease. When cells were treated with 10-fold increased UA (10 µM), MMP and ATP tended to be decreased, which may hint at cytotoxicity in higher concentrations. This however must not necessarily be the case as Ahsan and Cásedas et al. used a range of 3–30 µM UA on differentiated N2a cells and reported no cytotoxicity [32,33] and González-Sarrías et al. who also used SY5Y cells reported no cytotoxicity after 6–48 h or treatment with 10 µM UA [34]. In a different model, Ryu et al. found a significantly prolonged lifespan of nematodes treated with 50 µM UA [27] despite a loss of MMP. Authors explained that the reported loss of MMP may correlated with increased mitophagy [27].

Low micromolar UA concentrations, provided significant protection against H_2_O_2_-induced ROS and increased antioxidant enzyme activity [33] and nanomolar concentrations helped to maintain calcium homeostasis in SH-SY5Y cells [36]. Although we generally found increased cellular ROS in SY5Y-APP695, UA did not decrease basal ROS levels in either cell line. Inhibition of complex-I by rotenone, leading to increased ROS [49] resulted in a drop of MMP and ATP in both cell lines. This however was not attenuated by either 1 µM or 10 µM UA. On the contrary, in some cases (1 µM and 10 µM on ATP of SY5Ymock and 10 µM on MMP of SY5Y-APP695) UA even added to the effect of rotenone. One way in which this might be explained, is that UA specifically affects CIII activity. This could impair the electron transport to CIV. Another theory might stem from UA’s effect on the gene expression of NDUFV1, which encodes a part of CI. This increase might suggest higher coverage of CI in the membranes resulting in a stronger response to rotenone. In general, the observed increases in OXPHOS-related gene expression could indicate a delayed response of the cells towards UA’s effect on them. Therefore, based on our data of SY5Y cells, we are unable to confirm UA’s effect as an antioxidant, which has been reported elsewhere [33,50,51], but instead the results could point towards a hormetic response of the cell towards UA exposure.

Another mitochondrial parameter, mitochondrial mass, was also investigated in both cell lines. Since respiration tended to be lower in SY5Y-APP695 cells treated with UA, we investigated if this was due to reduced mitochondrial content. Citrate synthase activity, a common marker for mitochondrial mass [42,43] was not effected by UA treatment. However, gene expression of markers for mitochondrial biogenesis such as CREB and TFAM were increased specifically in SY5Y-APP695 cells following UA treatment. Another marker, PGC1α showed no apparent changes following UA exposure, and CS expression, although significantly increased in SY5Ymock, showed only trends in the disease model. This suggests that effects to mitochondrial biogenesis might only occur after prolonged exposure. Toney et al. found signs for enhanced mitochondrial biogenesis in bone marrow-derived macrophages in form of increased PGC1α and SIRT1 levels after pretreatment with 30 µM UA for 12 h but generally, little is known about UA’s effect on mitochondrial biogenesis in neuronal cells [38]. Here, gene expression of PGC1α and SIRT1 were more in accordance with our results suggesting no changes to mitochondrial mass, as both markers were unaffected in all groups with SIRT1 only tending to be increased after UA treatment. However, PGC1a protein and its activation has been described to be very short-lived [52]. For this reason, the lack of change to its gene expression should be considered carefully, as it could have been increased only for a very short time activating transcription of genes downstream, like NRF1, GABPα, ESRRα, and ESRRγ [53]. In a clinical trial by Andreux et al. subjects treated with 1000 mg/day UA for 28 days and showed trends for improved mitochondrial function, as well a significantly increased PGC1α expression in skeletal muscle [37]. Ryu et al. found an initial reduction in mitochondrial content on day 1 after treatment of *C. elegans* with UA, which later turned into an increase on day 10, which would again point towards a delayed hormetic reaction of the study model.

Looking at the PCR results in general, data suggests that expression of NRF1, COX5A, and ATP5D was increased in SY5Y-APP695 cells. NRF1 is a key inducer for the transcription cyclooxygenases and is therefore in agreement with the increase seen in COX5A. Manczak et al. also found that CIV and CV expression increased during the progression of AD in brain sections of patients with early and manifested AD [54]. While we were unable to see changes to CI expression, Manczak et al., reported a decrease in the transcription of CI [54]. An explanation for this could be the decreased ATP and MMP levels of SY5Y-APP695, or generally the decreased respiration, which could cause a response on genetic level and an increase in CIV and CV transcription. Treatment of SY5Y-APP695 cells with urolithin tended to reduce complex activities while endogenous respiration was virtually identical to untreated cells. Furthermore, the treatment showed no effect on the respiration of SY5Ymock cells. Still, in both cell lines expression of genes related to mitochondrial biogenesis, and thus OXPHOS, increased after UA treatment.

As mentioned before, TFAM, a key player in mitochondrial biogenesis, was significantly and specifically increased in SY5Y-APP695 cells. Similarly CREB, which is known to activate the PGC1α-NRF1-TFAM pathway [55,56], was also increased specifically in SY5Y-APP695 cells. However, both PGC1α and NRF1 were unaffected by UA in this cell line, suggesting that either both were only increased for a short period of time or that CREB might induce TFAM gene transcription via a different signal way. Although most pathways include members of the PGC family, Singh et al. have reported that TFAM expression was increased in a ESRRα-dependent manner in the liver of C57BL/6 mice [57]. Furthermore, GA Binding Protein Transcription Factor Subunit Alpha (GABPα, is also known to be involved in mitochondrial biogenesis and activation of TFAM [58,59]. We found an increase of both, ESRRα and GABPα in both cell lines. However, a model specific increase, similar to TFAM’s increase in SY5Y-APP695 cells, was only found for ESRRγ. Wang et al. found that ESRRα and ESRRγ knockout led to drastic reduction in the expression of not only GABPα and TFAM, but also of CS, CI, CIV, and CV expression in hearts of C57BL/6 mice [60]. It is therefore possible that treatment with UA might induce hormesis in an ESRR-dependent manner.

Little is known about Urolithin’s effect on ESRR gene expression and its implications. Next to increased PGC1α gene expression, Andreux et al. also found that 1000 mg/day UA led to significant increases of ESRRα and significantly higher mitochondrial mass in subjects’ skeletal muscle [37]. In skeletal muscle myoblasts of C57BL/6 mice treated with 10 mg/kg bodyweight UA, Ghosh et al. found increased PGC1α and SIRT1 expression, as well as increased ATP levels [39]. Since it is not unambiguously clear in which way PGC1a was or was not affected due to its short half-life, alternatives should be considered. One possible alternative might be the involvement of another member of the PGC family, PGC1β. Di Shao et al., for example reported ESRRα and PGC1β mediated mitochondrial biogenesis in C2C12 myoblasts [61].

Finally, changes to increased mitochondrial biogenesis after UA treatment could lead to the observed changes in CI and CS transcription. In this way, the dysregulated overexpression of CIV and CV in SY5Y-APP695 cells might actually be rebalanced for the generation of new mitochondria. Furthermore, although it is difficult to translate the results from Manczak et al., to our cellular model, increased expression of CI could also counteract the decline which might occur in a more advanced stage of our disease-model. In this manner, UA might exhibit neuroprotective effects in this cellular model, not by bringing SY5Y-APP695 specific changes back in line with SY5Ymock, but instead by producing a hormetic response rebalancing gene transcription by upregulation of genes not affected in the disease model. 

Recently, it was discovered that one mechanism behind of urolithin’s neuroprotections might be its ability to modulate autophagic processes. Ryu et al. provided evidence of increased longevity in *C. elegans* associated with generally improved mitochondrial function and mitophagy [27]. Nevertheless, UA’s effects vary depending on the model used for the study. While UA was found to improve mitophagy in *C. elegans*, Ahsan et al. found this process to be unaltered in cortical neuron of mice. Instead, general autophagy was enhanced [32]. This is in agreement with other studies providing evidence for enhanced autophagy, like Zhang et al., showed for MIN6β cells and the pancreas of mice [31]. Zhao et al. found increased autophagy in sw620 CRC cells which was induced by treatment with 1.5 µM UA for 24 h [30], while apoptosis only occurred at concentrations starting from 30 µM. 

Here, UA was able to increase gene expression of MAP1LC3 as well as increase fluorescence of a marker dye specifically binding to autophagosomes in SY5Y-APP695 cells. Roushop et al. described that impaired induction of MAP1LC3 gene expression could lead to a depletion of LC3 protein, halting autophagy in a state of hypoxia [62]. Swaminathan et al. also suggested that LC3-associated autophagy was impaired if MAP1LC3 gene was downregulated hindering in carotid plaques of subjects [63]. Furthermore, we found higher gene expression of SQSTM1/p62 in SY5Y-APP695 compared to SY5Ymock. González-Rodríguez et al., who investigated autophagy in liver diseases, also reported an increase in SQSTM1/p62 expression in samples with impaired autophagy [64]. Here, treatment with UA showed a trend to reduce SQSTM1/p62 expression to a similar level found in treated SY5Ymock. A similar pattern was also found by Zhang et al., reporting both, upregulated LC3 and downregulated SQSTM1/p62 in pancreatic MIN6β cells [31]. Although this generally points to increased autophagy, results of the protein levels of LC3B-II, the phosphatidylethanolamine-conjugated form of LC3B-I, were unaffected in both cell lines. During autophagy, LC3B-I is converted to LC3B-II and binds to autophagosomal membranes. Increased formation of autophagosomes was suggested by our initial tests using a fluorescence dye labeling acidic autophagosomes. Nevertheless, these results need to be considered with caution, as this increase could also result from increased autophagosome synthesis or decreased autophagosome turnover [65]. The same is true for results of LC3B-II protein alone. Therefore, another protein marker for autophagy, p62/sequestosome 1, which binds to poly-ubiquitinated proteins and LC3B-II, effectively marking them during the autophagic process for degradation. In this sense, high autophagic flux should be represented by lower p62 levels [65]. Naturally, since p62 itself can also be affected by e.g., oxidative stress [65,66], changes to it have to be considered with care as well. Here, data did not show changes to LC3B-II or p62 protein levels suggesting no effect of 1 µM UA on SY5Y cells. Since UA did not affect cellular ROS, changes to p62 due to different levels of oxidative stressors should not be relevant. Increased fluorescence of the autophagosome-binding dye could also stem from it not only binding to autophagosomes, but also lysosomes or other acidic compartments [67]. Considering results of the gene expression, Mizushima et al. report that it has not yet been shown whether autophagic activity is transcriptionally upregulated [67]. Son and Zhou et al., reported that autophagy could also assist in the degradation of amyloid beta protein [68,69]. However, as expected considering the results for autophagy, assessment of Aβ_1–40_ levels in SY5Y-APP695 cells showed was not affected by UA treatment. 

Taken together, treatment of SY5Y cells with 1 µM UA did not alter markers for autophagy in any capacity. 

## 4. Materials and Methods

### 4.1. Chemicals

All chemicals used were of the highest purity available and purchased from either Sigma Aldrich, Merck or VWR. Urolithin A (HY-100599) was aquired from MedChemExpress (Monmouth Junction, NJ, USA). Type-1 ultrapure water was used to prepare aqueous solutions.

### 4.2. Cells

In this study, two variants of the neuroblastoma SH-SY5Y cell line were used. Cells stably transfected with the human APP695 coding region served as established model for early AD, while cells transfected with the corresponding empty pCEP4 vector were used as controls [41].

SY5Ymock and SY5Y-APP695 were grown in 250 mL Greiner flasks with Dulbecco’s modified Eagle medium (DMEM) (Gibco, Thermo Scientific) supplemented with 10% (*v*/*v*) fetal bovine serum (FBS), 1% MEM-vitamins, pyruvate, and nonessential amino acids and antibiotics (penicillin, streptomycin). For selectivity, 3 µg/mL hygromycin B were added to the medium. When cells’ growth reached a confluency of 70–80%, cells were transferred to a new culture flask. 

For experiments, cells were harvested from Greiner flasks, counted using a Neubauer Chamber and diluted to yield a cell suspension of 10^6^ cells/mL. Cells were then sown into 6-well plates (qPCR, Western Blot 50^5^ cells/well), 24-well plates (MMP, 10^5^ cells/well), or 96-well plates (ATP, Autophagy and ROS assays, 2 × 10^4^ cells/well) depending on the experiment that was performed. Cells were seeded in reduced DMEM (2% FBS & other supplements identical to cultivating medium) and allowed to attach to the bottom of the wells for 48 h before being exposed to 1 µM to 10 µM Urolithin A (UA). UA was prepared in DMSO. Its final concentration in all experiments ranged from 0.1 to 1 %. In MMP and ATP experiments, UA’s effect on complex I inhibition was also investigated. For this, cells were incubated with 25 µM Rotenone 1 h after UA exposure. All experiments were conducted after a total incubation time of 24 h incubation.

### 4.3. Autophagy

Previously seeded cells were incubated with UA or control for 24 h to assess autophagy using an autophagy assay kit (Sigma Aldrich, Munich, Germany). Medium was removed from cells and autophagy detection reagent was added to each well. The microplate was incubated for 1 h at 37 °C and 5% CO_2_. The microplate was centrifuged at 300× *g* for 5 min. The autophagy detection reagent was removed and cells were washed with washing buffer. This step was repeated thrice, before autophagy was measured using a ClarioStar plate reader (BMG Labtech, Ortenberg, Germany) and an excitation wavelength of 360 nm and an emission wavelength of 520 nm. According to the manufacturer the fluorescence dye in this assay kit is of proprietary design and based on the accumulation in acidic organelles.

### 4.4. Western Blotting

Samples (15 µg protein) were prepared using 20 mM Tris buffer (pH 7.4), Laemmli Sample Buffer 2× (Bio-Rad, Munich, Germany), and β-mercaptoethanol. After denaturation for 5 min at 95 °C, the samples were loaded on a 12.5% acrylamide gel and separated by SDS-PAGE for 40 min and 150 V. Gels were transferred onto a polyvinylidene fluoride (PVDF) membrane (60 min at 300 mA) at 4 °C. After the transfer, the membrane was treated with skim milk blocking solution for 30 min and then washed three times with Tris-buffered saline containing Tween^®^20 (TBST). The membrane was then treated with primary antibodies overnight at 4 °C with constant shaking. Following incubation, the membrane was washed with TBST three times and incubated with horseradish peroxidase (HRP)-conjugated secondary antibody (Calbiochem via Merck Millipore, Darmstadt, Germany) for 10 min at room temperature with constant shaking. The membrane was then washed three times with TBST and treated with Luminata™ Western HRP Substrate (Merck Millipore, Darmstadt, Germany) for visualization. Band analysis was performed using a ChemiDoc XRS system (Bio-Rad, Munich, Germany). LC3B-I/II and p62 protein were detected using LC3B Antibody and SQSTM1/p62 Antibody (#2775S & #5114; Cell Signaling Technology, Danvers, MA, USA). Actin-β antibody (MAB1501; Merck Millipore, Darmstadt, Germany) was used to verify equal protein loading. Standard used for blotting was Precision Plus Protein™ All Blue Prestained Protein Standards (Bio-Rad, Munich, Germany).

### 4.5. High-Resolution Respirometry

Oxygraph-2k respirometer (Oroboros, Innsbruck, Austria) was used to investigate the respiration of cells as described earlier [70]. For data evaluation, the software DatLab v. 4.3.2.7 was used. Mitochondrial respiration was assessed using a protocol created by Gnaiger et al. [71] and includes controlled additions of substrates, inhibitors and uncouplers of the oxidative phosphorylation. Following the injection of cells into the Oxygraph 2k, cell membranes were disrupted with 8 mM digitonin to remove naive substrates. Then, complex-I (CI) activity was determined by addition of 5 mM glutamate and 2 mM malate (CI_L_). Addition of 2 mM ADP started ATP production at CV and reflects therefore physiological CI_P_ activity. Subsequent addition of 10 mM succinate activated CII, resulting in a state of physiological respiration CI + II_P_. Uncoupled CI + II_E_ was determined via stepwise addition of carbonyl cyanide *p*-(trifluoromethoxy) phenylhydrazone (FCCP, up to 1–1.5 µM). Uncoupled CII_E_ was measured following inhibition of complex-I via rotenone (0.5 µM). Leak respiration CII_L_ was measured after addition of oligomycin (2 µg/mL). After this, addition of 2.5 µM antimycin A resulted showed residual oxygen consumption, caused by enzymes that are not involved in OXPHOS. Finally, CIV_E_ activity was measured by addition of 0.5 mM tetramethylphenylenediamine (TMPD), an artificial substrate of complex IV and 2 mM ascorbate to regenerate TMPD. All data points were corrected for residual oxygen consumption and CIV_E_ was additionally corrected for autooxidation rate, determined via addition of excess NaN_3_ at the end of the experiment. A subset of the cells measured was frozen at −80 °C for later determination of protein content.

### 4.6. Citrate Synthase Activity

Cell culture flasks with a confluency of 70% were incubated with UA for 24 h. Then, medium was removed and cells were washed with PBS. Cells were rinsed of the cell culture flask with 10 mL mitochondrial respiration medium (MiR05 [17]) and centrifuged at 300× *g* for 5 min. Then, cells were counted using a Neubauer Chamber and diluted to yield a cell suspension of 10^6^ cells/mL. Samples were frozen in LN2 and stored at −80 °C until experiments were conducted. To determine citrate synthase activity, as a marker for mitochondrial content, the samples were allowed to thaw while a reaction medium (0.1 mM 5,5′-dithio-bis-(2-nitrobenzoic acid) (DTNB), 50 µM EDTA, 0.31 mM acetyl coenzyme A, 5 mM triethanolamine hydrochloride, and 0.1 M Tris-HCl) was mixed. 40 µL of the samples were added to a 96-well plate bevor 110 µL of the reaction medium were added. The microplate and the starting reagent (0.5 mM oxaloacetate) were temperated to 30 °C for 5 min. Thereafter, 50 µL of the starting reagent were added to each well of the microplate. Absorbance was measured in a ClarioStar plate reader (BMG Labtech, Ortenberg, Germany) at wavelength 412 nm.

### 4.7. Protein Content

Previously frozen cells from CS experiments were thawed and protein contents were determined using a Pierce BCA Protein Assay Kit (Thermo Fisher Scientific, Waltham, MA, USA) according to the manufacturer’s instructions. Absorbance was measured using a ClarioStar plate reader (BMG Labtech, Ortenberg, Germany).

### 4.8. ROS Determination

Cellular ROS was determined via DCFDA/H_2_DCFDA reaction using a cellular ROS assay kit (Abcam, Berlin, Germany) according to the manufacturer’s guidelines. Prior to the experiment, cells were incubated with 1 µM UA or control for 24 h at 37 °C and 5% CO_2_. Fluorescence of samples was measured using a ClarioStar plate reader (BMG Labtech, Ortenberg, Germany) at an excitation wavelength of 485 nm and an emission wavelength of 535 nm.

### 4.9. Mitochondrial Membrane Potential (MMP)

Fluorescence dye rhodamine-123 (R123) was used to investigate MMP. For this, cells were seeded into 24-well plates and incubated with 1 µM or 10 µM UA for 24 h. After incubation, 0.4 µM R123 were added to the cells at 37 °C and 5% CO_2_ for 15 min. Subsequently, cells were centrifuged at 300× *g* for 5 min. before medium was removed and cells were washed with HBSS buffer (supplemented with Mg^2+^, Ca^2+^ and HEPES; pH 7.4; 37 °C) to remove excess dye. Cells were resuspended in fresh HBSS buffer and R123 fluorescence was determined with a ClarioStar plate reader (BMG Labtech, Ortenberg, Germany) at an excitation wavelength of 490 nm and an emission wavelength of 535 nm.

### 4.10. Adenosine Triphosphate Levels

Adenosine triphosphate (ATP) concentrations were determined using an ATPlite Luminescence Assay System (Perkin Elmer, Rodgau-Jügesheim, Germany). Cells were incubated in 96-well plates and treated with 1 µM or 10 µM UA for 24 h. The 96-well plate was allowed to cool to room temperature for 10 min and then ATP concentration was assessed following the manufacturer’s instructions. The emitted light was measured with a ClarioStar plate reader (BMG Labtech, Ortenberg, Germany). Due to a linear relation between emitted light and ATP production, unknown sample concentrations were calculated via linear regression of a standard curve. 

### 4.11. Aβ_1–40_ Concentrations

Concentrations of Aβ_1–40_ were determined using Amyloid beta1–40 Kit (Cisbio) according to the manufacturer’s instructions. Cells were previously seeded in culture flasks until they reached a confluency of 70–80%, then incubated with 1 µM UA or ctrl. Cells were harvested 24 h later, washed with PBS once, and stored in PBS containing complete^TM^, EDTA-free protease inhibitor cocktail (Sigma-Aldrich) at −80 °C until experimentation. Upon thawing the samples, cells were lysed using Cell Extraction Buffer (Invitrogen) before applying the kit’s protocol. Fluorescence was measured using a ClarioStar plate reader with HTRF filters (BMG Labtech) at an emission wavelength of 665 nm for the acceptor and 620 nm for the donor. Samples were measured in triplicates.

### 4.12. Quantitative Real-Time PCR (qRT-PCR)

Cells previously seeded in a 6-well microplate were rinsed with PBS once and then washed off the plate. Cells were centrifuged at 300× *g* for 5 min and then resuspended in 1 mL RNAlater (Qiagen, Hilden, Germany), frozen in LN_2_ and stored at −80 °C until experimentation. Total RNA was isolated from cell homogenate using the RNeasy Mini Kit (Qiagen, Hilden, Germany) according to the manufacturer’s instructions.

Purity and quantification was estimated using a NanoDrop 2000× spectrophotometer (Thermo-Fisher Scientific, Waltham, MA, USA). To remove residual gDNA, RNA was treated with TurboDNA free Kit (Qiagen, Hilden, Germany). cDNA was synthesized from 1 µg RNA using iScript cDNA synthesis kit (BioRad, Munich, Germany) according to manufacturer’s instructions. Quantitative real-time PCR was performed on a CFX96 Touch real-time PCR detection system (BioRad, Munich, Germany) using SYBR Green technology. Each sample was analyzed in triplicates with a total sample volume of 10 µL. Primer sequences, concentrations, product sizes and PCR conditions can be found in Table 2. Gene expression was analyzed using the 2(-∆∆Cq) method using BioRad CFX manager (BioRad, Munich, Germany) and normalized to Phosphoglycerate Kinase 1 (PGK1), beta-actin (ACTβ) and Glyceraldehyde 3-phosphate dehydrogenase (GAPDH) expression levels according to the MIQE guidelines [72]. Primers for qPCR were acquired from Biomol Hamburg, Germany) or Sigma-Aldrich (Munich, Germany).

### 4.13. Data Handling and Statistics

Unless stated otherwise, data is presented as mean ± SEM. Outliers were removed via ROUT (q = 1%) outlier test. Statistical testing was performed using two-way ANOVA defining treatment as factor 1 and cell line as factor 2. All groups were compared to each other and results were corrected by a false discovery rate correction according to Benjamini, Krieger, and Yekutieli.

All statistical analysis was carried out with GraphPad Prism (GraphPad Prism version 8.2, GraphPad Software, San Diego, CA, USA). 

## 5. Conclusions

In conclusion, the results of this study further suggest that UA might counteract the model-specific decrease of mitochondrial function. Mitochondrial function was largely unaffected by 1 µM UA but showed trends to reduce OXPHOS. Increased UA concentrations of 10 µM already led to reduced MMP, ATP. Data suggests that UA did not affect autophagy neither SY5Ymock nor SY5Y-APP695 cells. In addition, the modulation of gene transcription for mitochondrial biogenesis and OXPHOS possibly suggests a hormetic effect induced by UA. 

## Figures and Tables

**Table 1:** Aβ_1–40_ levels in SY5Y-APP695 cells treated with 1 µM UA or control (ctrl). Aβ was determined via Homogeneous Time Resolved Fluorescence (HTRF). Data is adjusted to protein content of the samples. Data is displayed as mean ± SEM. N = 10. Statistical significance was tested via Student’s *t*-test. Statistical differences are given as ns *p* > 0.05.

	SY5Y-APP695		
	Ctrl	1 µM UA	*p*
Aβ_1–40_ [pg/mg_Protein_]	53.97 ± 3.53	53.84 ± 2.15	0.98; ns

**Table 2:** Primer sequences, manufacturer’s, product sizes, concentrations and program used for qRT-PCR measurement. ACTβ, PGK1, and GAPDH were used as housekeeping genes.

Primer	Sequence	Size [bp]	Conc [µM]	Annealing Temp. (Time) (Cycle No.)
ACTβ	5′-GGACTTCGAGCAAGAGATGG-3′ 5′-AGCACTGTGTTGGCGTACAG-3′	234	0.2	58 °C (30 s), (45×)
PGK1	5′-CTGTGGGGGTATTTGAATGG-3′ 5′-CTTCCAGGAGCTCCAAACTG-3′	198	0.2	58 °C (30 s), (45×)
GAPDH	5′-GAGTCAACGGATTTGGTCGT-3′ 5′-TTGATTTTGGAGGGATCTCG-3′	238	0.2	58 °C (30 s), (45×)
CS	5′-GGGTCTGATGAAGTTTGTGG-3′ 5′-GATTAGGGAAGAAGGGACCA-3′	221	0.4	58 °C (30 s), (45×)
NDUVF1 (CI)	5′-GCAGAAGAAGGCCATACGA-3′ 5′-CTCGCTTTATTGTCCAGCAT-3′	206	0.2	58 °C (30 s), (45×)
COX5A (CIV)	5′-GCATGCAGACGGTTAAATGA-3′ 5′-AGTTCCTCCGGAGTGGAGAT-3′	152	0.2	58 °C (45 s), (45×)
ATP5D (CV)	5′-CAACCAGATGTCCTTCACCT-3′ 5′-AACAACTGCACCGAAGAGTC-3′	240	0.2	58 °C (45 s), (45×)
MAP1LC3	5′-AGCAGCATCCAACCAAAATC-3′ 5′-CTGTGTCCGTTCACCAACAG-3′	187	0.1	58 °C (45 s), (45×)
SQSTM1	5′-CACCTCTCTGAGGGCTTCTC-3′ 5′AGTTTCCTGGTGGACCCATT-3′	97	0.2	58 °C (45 s), (45×)
SIRT1	5′-TGTGGTAGAGCTTGCATTGA-3′ 5′GCCTGTTGCTCTCCTCATTA-3′	153	02.	58 °C (45 s), (45×)
CREB1	5′-TGGAGTTGTTATGGCATCCT-3′ 5′-ATTTTCAAGCACTGCCACTC-3′	169	0.2	58 °C (45 s), (45×)
PGC1α	5′-CATCCCTCTGTCATCCTC-3′ 5′-GCAGACCTAGATTCAAACTC-3′	146	0.2	60 °C (30 s), (45×)
ESRRα	5′-CCAATTCAGACTCTGTGC-3′ 5′-CTTCATACTCCAGCAGGG-3′	87	0.45	58 °C (30 s), (45×)
ESRRγ	5′-GTGATGTGTACCATACTGTG-3′ 5′-TTAGCAGTCAAAAGTGGAAG-3′	198	0.45	58 °C (30 s), (45×)
NRF1	5′-GTAACCCTGATGGCACTGTC-3′ 5′-TCTGGATGGTCATCTCACT-3′	183	0.2	58 °C (45 s), (45×)
GABPα (NRF2)	5′-CAACCTGTGCAAATTATTCC-3′ 5′-GGTGAGCTTCTATCTTCTCC-3′	131	0.45	58 °C (30 s), (45×)
TFAM	5′-TCCCCCTTCAGTTTTGTGTA-3′ 5′-ATCAGGAAGTTCCCTCCAAC-3′	189	0.4	58 °C (30 s), (45×)

## Data Availability

The dataset generated during this study is available from the corresponding author upon reasonable request.

## Supplementary Materials

The following are available online at https://www.mdpi.com/article/10.3390/ijms22158333/s1.
