# Supplementary material for: Effects of Urolithin A on Mitochondrial Parameters in a Cellular Model of Early Alzheimer Disease
Source: Int J Mol Sci. 2021 Aug 3;22(15):8333. doi: 10.3390/ijms22158333 (PMC8347929; doi:10.3390/ijms22158333)
Supplement: Supplementary file 1 [file ijms-22-08333-s001.zip › ijms-1296767-supplementary.pdf]

Supplementary Table S1:

Statistical parameters for figures 2-5 and table 1 in the manuscript. A: Respiration of cells (Figure 2). B: Mitochondrial Membrane potential (Figure 2&3). C: Adenosine triphosphate (ATP) levels (Figure 2&3). D: Citrate synthase activity (Figure 3). E: Cellular ROS (Figure 3). F: $A\beta_{1-40}$ levels of cells (Table 1). G: Results for qRT-PCR (Figure 4&5) H: Autophagy experiments (Figure 5) I: Western Blot experiments (Figure 2). Statistical evaluation was done Two-way ANOVA with false discovery rate correction according to Benjamini, Krieger and Yekutieli in A-H and student's t-test in I). Displayed are the adjusted p-values. Shown are mean ± SEM. Statistical significance was given at $^{ns}p > 0.05$; $*p < 0.05$; $**p < 0.01$; $***p < 0.001$; $****p < 0.0001$.

**A) Respiration**		**Individual p value**	**Adjusted p value (FDR corrected)**
endogen	ctrl:SY5Ymock vs. ctrl:SY5Yapp	< 0.0001	< 0.0001 ****
	ctrl:SY5Ymock vs. UA 1µM:SY5Ymock	0.7682	0.2843 ns
	ctrl:SY5Ymock vs. UA 1µM:SY5Yapp	< 0.0001	< 0.0001 ****
	ctrl:SY5Yapp vs. UA 1µM:SY5Ymock	< 0.0001	< 0.0001 ****
	ctrl:SY5Yapp vs. UA 1µM:SY5Yapp	0.8123	0.2843 ns
	UA 1µM:SY5Ymock vs. UA 1µM:SY5Yapp	< 0.0001	< 0.0001 ****
digitonine	ctrl:SY5Ymock vs. ctrl:SY5Yapp	0.0014	0.0010 **
	ctrl:SY5Ymock vs. UA 1µM:SY5Ymock	0.4338	0.1518 ns
	ctrl:SY5Ymock vs. UA 1µM:SY5Yapp	< 0.0001	< 0.0001 ****
	ctrl:SY5Yapp vs. UA 1µM:SY5Ymock	0.0108	0.0057 **
	ctrl:SY5Yapp vs. UA 1µM:SY5Yapp	0.3853	0.1518 ns
	UA 1µM:SY5Ymock vs. UA 1µM:SY5Yapp	0.0005	0.0006 ***
CI (L)	ctrl:SY5Ymock vs. ctrl:SY5Yapp	< 0.0001	< 0.0001 ****
	ctrl:SY5Ymock vs. UA 1µM:SY5Ymock	0.6873	0.2405 ns
	ctrl:SY5Ymock vs. UA 1µM:SY5Yapp	< 0.0001	< 0.0001 ****
	ctrl:SY5Yapp vs. UA 1µM:SY5Ymock	< 0.0001	< 0.0001 ****
	ctrl:SY5Yapp vs. UA 1µM:SY5Yapp	0.6101	0.2405 ns
	UA 1µM:SY5Ymock vs. UA 1µM:SY5Yapp	< 0.0001	< 0.0001 ****
CI (P)	ctrl:SY5Ymock vs. ctrl:SY5Yapp	0.0145	0.0153 *
	ctrl:SY5Ymock vs. UA 1µM:SY5Ymock	0.3816	0.2004 ns
	ctrl:SY5Ymock vs. UA 1µM:SY5Yapp	0.0005	0.0017 **
	ctrl:SY5Yapp vs. UA 1µM:SY5Ymock	0.1034	0.0814 ns
	ctrl:SY5Yapp vs. UA 1µM:SY5Yapp	0.2954	0.1861 ns
	UA 1µM:SY5Ymock vs. UA 1µM:SY5Yapp	0.0074	0.0116 *
CI & CII (P)	ctrl:SY5Ymock vs. ctrl:SY5Yapp	0.0017	0.0012 **
	ctrl:SY5Ymock vs. UA 1µM:SY5Ymock	0.5171	0.1810 ns
	ctrl:SY5Ymock vs. UA 1µM:SY5Yapp	< 0.0001	0.0001 ***
	ctrl:SY5Yapp vs. UA 1µM:SY5Ymock	0.0110	0.0058 **
	ctrl:SY5Yapp vs. UA 1µM:SY5Yapp	0.3390	0.1424 ns
	UA 1µM:SY5Ymock vs. UA 1µM:SY5Yapp	0.0005	0.0006 ***
CI & CII (U)	ctrl:SY5Ymock vs. ctrl:SY5Yapp	< 0.0001	< 0.0001 ****
	ctrl:SY5Ymock vs. UA 1µM:SY5Ymock	0.4455	0.1559 ns
	ctrl:SY5Ymock vs. UA 1µM:SY5Yapp	< 0.0001	< 0.0001 ****
	ctrl:SY5Yapp vs. UA 1µM:SY5Ymock	< 0.0001	< 0.0001 ****
	ctrl:SY5Yapp vs. UA 1µM:SY5Yapp	0.4300	0.1559 ns
	UA 1µM:SY5Ymock vs. UA 1µM:SY5Yapp	< 0.0001	< 0.0001 ****
CII (U)	ctrl:SY5Ymock vs. ctrl:SY5Yapp	0.0010	0.0007 ***
	ctrl:SY5Ymock vs. UA 1µM:SY5Ymock	0.5515	0.1930 ns
	ctrl:SY5Ymock vs. UA 1µM:SY5Yapp	< 0.0001	< 0.0001 ****
	ctrl:SY5Yapp vs. UA 1µM:SY5Ymock	0.0058	0.0031 **

	ctrl:SY5Yapp vs. UA 1µM:SY5Yapp	0.1045	0.0439 *
	UA 1µM:SY5Ymock vs. UA 1µM:SY5Yapp	< 0.0001	< 0.0001 ****
CII (L)	ctrl:SY5Ymock vs. ctrl:SY5Yapp	0.0161	0.0169 *
	ctrl:SY5Ymock vs. UA 1µM:SY5Ymock	0.5296	0.2780 ns
	ctrl:SY5Ymock vs. UA 1µM:SY5Yapp	0.0003	0.0009 ***
	ctrl:SY5Yapp vs. UA 1µM:SY5Ymock	0.0716	0.0563 ns
	ctrl:SY5Yapp vs. UA 1µM:SY5Yapp	0.1969	0.1241 ns
	UA 1µM:SY5Ymock vs. UA 1µM:SY5Yapp	0.0023	0.0037 **
CIV (U)	ctrl:SY5Ymock vs. ctrl:SY5Yapp	0.0023	0.0016 **
	ctrl:SY5Ymock vs. UA 1µM:SY5Ymock	0.7392	0.2587 ns
	ctrl:SY5Ymock vs. UA 1µM:SY5Yapp	< 0.0001	< 0.0001 ****
	ctrl:SY5Yapp vs. UA 1µM:SY5Ymock	0.0063	0.0033 **
	ctrl:SY5Yapp vs. UA 1µM:SY5Yapp	0.1009	0.0424 *
	UA 1µM:SY5Ymock vs. UA 1µM:SY5Yapp	< 0.0001	< 0.0001 ****

**B) MMP**		**Individual p value**	**Adjusted p value (FDR corrected)**
UA 1 µM	ctrl:SY5Ymock vs. ctrl:SY5Yapp	0.0019	0.0013 **
	ctrl:SY5Ymock vs. UA 1 µM:SY5Ymock	0.7767	0.2718 ns
	ctrl:SY5Ymock vs. UA 1 µM:SY5Yapp	0.0003	0.0007 ***
	ctrl:SY5Yapp vs. UA 1 µM:SY5Ymock	0.0044	0.0023 **
	ctrl:SY5Yapp vs. UA 1 µM:SY5Yapp	0.5166	0.2170 ns
	UA 1 µM:SY5Ymock vs. UA 1 µM:SY5Yapp	0.0008	0.0009 ***
UA 10 µM	ctrl10:SY5Ymock vs. ctrl10:SY5Yapp	< 0.0001	< 0.0001 ****
	ctrl10:SY5Ymock vs. UA 10 µM:SY5Ymock	0.3279	0.1148 ns
	ctrl10:SY5Ymock vs. UA 10 µM:SY5Yapp	< 0.0001	< 0.0001 ****
	ctrl10:SY5Yapp vs. UA 10 µM:SY5Ymock	< 0.0001	< 0.0001 ****
	ctrl10:SY5Yapp vs. UA 10 µM:SY5Yapp	0.0770	0.0323 *
	UA 10 µM:SY5Ymock vs. UA 10 µM:SY5Yapp	< 0.0001	< 0.0001 ****
UA 1 µM Rotenone	ctrl:SY5Ymock vs. ctrl:SY5Yapp	0.0007	0.0007 ***
	ctrl:SY5Ymock vs. UA 1 µM:SY5Ymock	0.2527	0.1061 ns
	ctrl:SY5Ymock vs. UA 1 µM:SY5Yapp	0.0003	0.0005 ***
	ctrl:SY5Yapp vs. UA 1 µM:SY5Ymock	0.0185	0.0097 **
	ctrl:SY5Yapp vs. UA 1 µM:SY5Yapp	0.7205	0.2522 ns
	UA 1 µM:SY5Ymock vs. UA 1 µM:SY5Yapp	0.0081	0.0057 **
UA 10 µM Rotenone	ctrl:SY5Ymock vs. ctrl:SY5Yapp	< 0.0001	< 0.0001 ****
	ctrl:SY5Ymock vs. UA 10 µM:SY5Ymock	0.7271	0.2545 ns
	ctrl:SY5Ymock vs. UA 10 µM:SY5Yapp	< 0.0001	< 0.0001 ****
	ctrl:SY5Yapp vs. UA 10 µM:SY5Ymock	< 0.0001	< 0.0001 ****
	ctrl:SY5Yapp vs. UA 10 µM:SY5Yapp	0.0620	0.0260 *
	UA 10 µM:SY5Ymock vs. UA 10 µM:SY5Yapp	< 0.0001	< 0.0001 ****

**C) ATP**		**Individual p value**	**Adjusted p value (FDR corrected)**
UA 1 µM	ctrl:SY5Ymock vs. ctrl:SY5Yapp	< 0.0001	< 0.0001 ****
	ctrl:SY5Ymock vs. UA 1µM:SY5Ymock	0.7024	0.2503 ns
	ctrl:SY5Ymock vs. UA 1µM:SY5Yapp	< 0.0001	< 0.0001 ****
	ctrl:SY5Yapp vs. UA 1µM:SY5Ymock	< 0.0001	< 0.0001 ****
	ctrl:SY5Yapp vs. UA 1µM:SY5Yapp	0.7151	0.2503 ns
	UA 1µM:SY5Ymock vs. UA 1µM:SY5Yapp	< 0.0001	< 0.0001 ****
UA 10 µM	ctrl10:SY5Ymock vs. ctrl10:SY5Yapp	< 0.0001	< 0.0001 ****
	ctrl10:SY5Ymock vs. UA 10µM:SY5Ymock	0.1911	0.0803 ns

	ctrl10:SY5Ymock vs. UA 10µM:SY5Yapp	< 0.0001	< 0.0001 ****
	ctrl10:SY5Yapp vs. UA 10µM:SY5Ymock	0.0008	0.0004 ***
	ctrl10:SY5Yapp vs. UA 10µM:SY5Yapp	0.4310	0.1508 ns
	UA 10µM:SY5Ymock vs. UA 10µM:SY5Yapp	< 0.0001	< 0.0001 ****
UA 1 µM Rotenone	ctrl:SY5Ymock vs. ctrl:SY5Yapp	< 0.0001	< 0.0001 ****
	ctrl:SY5Ymock vs. UA 1µM:SY5Ymock	0.0803	0.0337 *
	ctrl:SY5Ymock vs. UA 1µM:SY5Yapp	< 0.0001	< 0.0001 ****
	ctrl:SY5Yapp vs. UA 1µM:SY5Ymock	< 0.0001	< 0.0001 ****
	ctrl:SY5Yapp vs. UA 1µM:SY5Yapp	0.8721	0.3053 ns
	UA 1µM:SY5Ymock vs. UA 1µM:SY5Yapp	< 0.0001	< 0.0001 ****
UA 10 µM Rotenone	ctrl10:SY5Ymock vs. ctrl10:SY5Yapp	< 0.0001	< 0.0001 ****
	ctrl10:SY5Ymock vs. UA 10µM:SY5Ymock	0.0006	0.0002 ***
	ctrl10:SY5Ymock vs. UA 10µM:SY5Yapp	< 0.0001	< 0.0001 ****
	ctrl10:SY5Yapp vs. UA 10µM:SY5Ymock	0.0010	0.0002 ***
	ctrl10:SY5Yapp vs. UA 10µM:SY5Yapp	0.4880	0.0854 ns
	UA 10µM:SY5Ymock vs. UA 10µM:SY5Yapp	0.0001	< 0.0001 ****

**D) Citrate Synthase activity**	**Individual p value**	**Adjusted p value (FDR corrected)**
ctrl:SY5Ymock vs. ctrl:SY5Yapp	0.0083	0.0522 ns
ctrl:SY5Ymock vs. UA 1µM:SY5Ymock	0.3283	0.4137 ns
ctrl:SY5Ymock vs. UA 1µM:SY5Yapp	0.0365	0.1150 ns
ctrl:SY5Yapp vs. UA 1µM:SY5Ymock	0.0628	0.1318 ns
ctrl:SY5Yapp vs. UA 1µM:SY5Yapp	0.4799	0.5039 ns
UA 1µM:SY5Ymock vs. UA 1µM:SY5Yapp	0.2219	0.3495 ns

**E) Cellular ROS assay**	**Individual p value**	**Adjusted p value (FDR corrected)**
ctrl:SY5Ymock vs. ctrl:SY5Yapp	0.0239	0.1364 ns
ctrl:SY5Ymock vs. UA 1µM:SY5Ymock	0.6658	0.7674 ns
ctrl:SY5Ymock vs. UA 1µM:SY5Yapp	0.0493	0.1364 ns
ctrl:SY5Yapp vs. UA 1µM:SY5Ymock	0.0650	0.1364 ns
ctrl:SY5Yapp vs. UA 1µM:SY5Yapp	0.7308	0.7674 ns
UA 1µM:SY5Ymock vs. UA 1µM:SY5Yapp	0.1230	0.1937 ns

**F) $A\beta_{1-40}$ HTRF assay**	**p value**
ctrl:SY5Yapp vs UA 1µM:SY5Yapp	0.9756

**G) qRT-PCR**		**Individual p value**	**Adjusted p value (FDR corrected)**
**CS**	ctrl:SY5Ymock vs. ctrl:SY5Yapp	0.6814	0.4770 ns
	ctrl:SY5Ymock vs. UA 1µM:SY5Ymock	0.0022	0.0046 **
	ctrl:SY5Ymock vs. UA 1µM:SY5Yapp	0.2201	0.1849 ns
	ctrl:SY5Yapp vs. UA 1µM:SY5Ymock	0.0005	0.0022 **
	ctrl:SY5Yapp vs. UA 1µM:SY5Yapp	0.1021	0.1072 ns
	UA 1µM:SY5Ymock vs. UA 1µM:SY5Yapp	0.0609	0.0853 ns
**NDUFV1**	ctrl:SY5Ymock vs. ctrl:SY5Yapp	0.4840	0.1751 ns
	ctrl:SY5Ymock vs. UA 1µM:SY5Ymock	0.0091	0.0048 **
	ctrl:SY5Ymock vs. UA 1µM:SY5Yapp	0.0025	0.0017 **
	ctrl:SY5Yapp vs. UA 1µM:SY5Ymock	0.0015	0.0016 **

	ctrl:SY5Yapp vs. UA 1µM:SY5Yapp	0.0004	0.0009 ***
	UA 1µM:SY5Ymock vs. UA 1µM:SY5Yapp	0.5003	0.1751 ns
**COX5A**	ctrl:SY5Ymock vs. ctrl:SY5Yapp	0.0006	0.0010 **
	ctrl:SY5Ymock vs. UA 1µM:SY5Ymock	0.4774	0.2005 ns
	ctrl:SY5Ymock vs. UA 1µM:SY5Yapp	0.0010	0.0010 **
	ctrl:SY5Yapp vs. UA 1µM:SY5Ymock	0.0033	0.0023 **
	ctrl:SY5Yapp vs. UA 1µM:SY5Yapp	0.8462	0.2962 ns
	UA 1µM:SY5Ymock vs. UA 1µM:SY5Yapp	0.0055	0.0029 **
**ATP5D**	ctrl:SY5Ymock vs. ctrl:SY5Yapp	0.0244	0.0128 *
	ctrl:SY5Ymock vs. UA 1µM:SY5Ymock	0.6081	0.2128 ns
	ctrl:SY5Ymock vs. UA 1µM:SY5Yapp	< 0.0001	< 0.0001 ****
	ctrl:SY5Yapp vs. UA 1µM:SY5Ymock	0.0750	0.0315 *
	ctrl:SY5Yapp vs. UA 1µM:SY5Yapp	0.0010	0.0007 ***
	UA 1µM:SY5Ymock vs. UA 1µM:SY5Yapp	< 0.0001	< 0.0001 ****
**SIRT1**	ctrl:SY5Ymock vs. ctrl:SY5Yapp	0.9382	0.9851 ns
	ctrl:SY5Ymock vs. UA 1µM:SY5Ymock	0.0451	0.0710 ns
	ctrl:SY5Ymock vs. UA 1µM:SY5Yapp	0.0235	0.0610 ns
	ctrl:SY5Yapp vs. UA 1µM:SY5Ymock	0.0290	0.0610 ns
	ctrl:SY5Yapp vs. UA 1µM:SY5Yapp	0.0145	0.0610 ns
	UA 1µM:SY5Ymock vs. UA 1µM:SY5Yapp	0.7188	0.9057 ns
**CREB**	ctrl:SY5Ymock vs. ctrl:SY5Yapp	0.1033	0.0813 ns
	ctrl:SY5Ymock vs. UA 1µM:SY5Ymock	0.5464	0.2869 ns
	ctrl:SY5Ymock vs. UA 1µM:SY5Yapp	< 0.0001	< 0.0001 ****
	ctrl:SY5Yapp vs. UA 1µM:SY5Ymock	0.2666	0.1680 ns
	ctrl:SY5Yapp vs. UA 1µM:SY5Yapp	< 0.0001	< 0.0001 ****
	UA 1µM:SY5Ymock vs. UA 1µM:SY5Yapp	< 0.0001	< 0.0001 ****
**PGC1α**	ctrl:SY5Ymock vs. ctrl:SY5Yapp	0.1961	0.5462 ns
	ctrl:SY5Ymock vs. UA 1µM:SY5Ymock	0.7984	> 0.9999 ns
	ctrl:SY5Ymock vs. UA 1µM:SY5Yapp	0.2378	0.5462 ns
	ctrl:SY5Yapp vs. UA 1µM:SY5Ymock	0.3016	0.5462 ns
	ctrl:SY5Yapp vs. UA 1µM:SY5Yapp	0.9938	> 0.9999 ns
	UA 1µM:SY5Ymock vs. UA 1µM:SY5Yapp	0.3468	0.5462
**NRF1**	ctrl:SY5Ymock vs. ctrl:SY5Yapp	< 0.0001	< 0.0001 ****
	ctrl:SY5Ymock vs. UA 1µM:SY5Ymock	0.1003	0.0421 *
	ctrl:SY5Ymock vs. UA 1µM:SY5Yapp	< 0.0001	< 0.0001 ****
	ctrl:SY5Yapp vs. UA 1µM:SY5Ymock	< 0.0001	< 0.0001 ****
	ctrl:SY5Yapp vs. UA 1µM:SY5Yapp	0.7545	0.2641 ns
	UA 1µM:SY5Ymock vs. UA 1µM:SY5Yapp	< 0.0001	< 0.0001 ****
**TFAM**	ctrl:SY5Ymock vs. ctrl:SY5Yapp	0.4029	0.3173 ns
	ctrl:SY5Ymock vs. UA 1µM:SY5Ymock	0.8860	0.4651 ns
	ctrl:SY5Ymock vs. UA 1µM:SY5Yapp	0.0169	0.0177 *
	ctrl:SY5Yapp vs. UA 1µM:SY5Ymock	0.5112	0.3220 ns
	ctrl:SY5Yapp vs. UA 1µM:SY5Yapp	0.0016	0.0051 **
	UA 1µM:SY5Ymock vs. UA 1µM:SY5Yapp	0.0147	0.0177 *
**GABPa**	ctrl:SY5Ymock vs. ctrl:SY5Yapp	0.8011	0.4206 ns
	ctrl:SY5Ymock vs. UA 1µM:SY5Ymock	0.0515	0.0406 *
	ctrl:SY5Ymock vs. UA 1µM:SY5Yapp	0.0009	0.0015 **
	ctrl:SY5Yapp vs. UA 1µM:SY5Ymock	0.0223	0.0234 *
	ctrl:SY5Yapp vs. UA 1µM:SY5Yapp	0.0003	0.0008 ***
	UA 1µM:SY5Ymock vs. UA 1µM:SY5Yapp	0.1041	0.0656 ns
**ESRRa**	ctrl:SY5Ymock vs. ctrl:SY5Yapp	0.1142	0.0720 ns
	ctrl:SY5Ymock vs. UA 1µM:SY5Ymock	0.0225	0.0236 *
	ctrl:SY5Ymock vs. UA 1µM:SY5Yapp	0.0001	0.0004 ***
	ctrl:SY5Yapp vs. UA 1µM:SY5Ymock	0.4342	0.2280 ns
	ctrl:SY5Yapp vs. UA 1µM:SY5Yapp	0.0089	0.0140 *
	UA 1µM:SY5Ymock vs. UA 1µM:SY5Yapp	0.0530	0.0418 *
**ESRRy**	ctrl:SY5Ymock vs. ctrl:SY5Yapp	0.2063	0.0831 ns
	ctrl:SY5Ymock vs. UA 1µM:SY5Ymock	0.2376	0.0831 ns
	ctrl:SY5Ymock vs. UA 1µM:SY5Yapp	< 0.0001	< 0.0001 ****

	ctrl:SY5Yapp vs. UA 1µM:SY5Ymock	0.0187	0.0098 **
	ctrl:SY5Yapp vs. UA 1µM:SY5Yapp	< 0.0001	< 0.0001 ****
	UA 1µM:SY5Ymock vs. UA 1µM:SY5Yapp	0.0012	0.0008 ***
**MAP1LC3**	ctrl:SY5Ymock vs. ctrl:SY5Yapp	0.1322	0.1198 ns
	ctrl:SY5Ymock vs. UA 1µM:SY5Ymock	0.1426	0.1198 ns
	ctrl:SY5Ymock vs. UA 1µM:SY5Yapp	0.1009	0.1198 ns
	ctrl:SY5Yapp vs. UA 1µM:SY5Ymock	0.0049	0.0104 *
	ctrl:SY5Yapp vs. UA 1µM:SY5Yapp	0.0031	0.0104 *
	UA 1µM:SY5Ymock vs. UA 1µM:SY5Yapp	0.8542	0.5979 ns
**SQSTM1**	ctrl:SY5Ymock vs. ctrl:SY5Yapp	0.0023	0.0122 *
	ctrl:SY5Ymock vs. UA 1µM:SY5Ymock	0.0230	0.0604 ns
	ctrl:SY5Ymock vs. UA 1µM:SY5Yapp	0.1601	0.2101 ns
	ctrl:SY5Yapp vs. UA 1µM:SY5Ymock	0.3717	0.3253 ns
	ctrl:SY5Yapp vs. UA 1µM:SY5Yapp	0.0733	0.1283 ns
	UA 1µM:SY5Ymock vs. UA 1µM:SY5Yapp	0.3534	0.3253 ns

**H) Autophagy assay kit**	**Individual p value**	**Adjusted p value (FDR corrected)**
UA 1µM:SY5Ymock vs. UA 1µM:SY5Yapp	< 0.0001	< 0.0001 ****
UA 1µM:SY5Ymock vs. ctrl :SY5Ymock	0.0851	0.0149 *
UA 1µM:SY5Ymock vs. ctrl :SY5Yapp	< 0.0001	< 0.0001 ****
UA 1µM:SY5Yapp vs. ctrl :SY5Ymock	< 0.0001	< 0.0001 ****
UA 1µM:SY5Yapp vs. ctrl :SY5Yapp	0.0124	0.0026 **
ctrl :SY5Ymock vs. ctrl :SY5Yapp	< 0.0001	< 0.0001 ****

**I) Western Blot**		**Individual p value**	**Adjusted p value (FDR corrected)**
**LC3B-I/β-Actin**	ctrl:SY5Ymock vs. ctrl:SY5Yapp	0.0656	0.4133 ns
	ctrl:SY5Ymock vs. UA 1µM:SY5Ymock	0.6648	0.6981 ns
	ctrl:SY5Ymock vs. UA 1µM:SY5Yapp	0.3593	0.5659 ns
	ctrl:SY5Yapp vs. UA 1µM:SY5Ymock	0.1515	0.4774 ns
	ctrl:SY5Yapp vs. UA 1µM:SY5Yapp	0.3362	0.5659 ns
	UA 1µM:SY5Ymock vs. UA 1µM:SY5Yapp	0.6256	0.6981 ns
**LC3B-II/β-Actin**	ctrl:SY5Ymock vs. ctrl:SY5Yapp	0.0264	0.0930 ns
	ctrl:SY5Ymock vs. UA 1µM:SY5Ymock	0.9595	> 0.9999 ns
	ctrl:SY5Ymock vs. UA 1µM:SY5Yapp	0.3904	0.5273 ns
	ctrl:SY5Yapp vs. UA 1µM:SY5Ymock	0.0295	0.0930 ns
	ctrl:SY5Yapp vs. UA 1µM:SY5Yapp	0.1520	0.3193 ns
	UA 1µM:SY5Ymock vs. UA 1µM:SY5Yapp	0.4185	0.5273 ns
**p62//β-Actin**	ctrl:SY5Ymock vs. ctrl:SY5Yapp	0.9035	0.9486 ns
	ctrl:SY5Ymock vs. UA 1µM:SY5Ymock	0.5618	0.8848 ns
	ctrl:SY5Ymock vs. UA 1µM:SY5Yapp	0.4712	0.8848 ns
	ctrl:SY5Yapp vs. UA 1µM:SY5Ymock	0.4840	0.8848 ns
	ctrl:SY5Yapp vs. UA 1µM:SY5Yapp	0.4012	0.8848 ns
	UA 1µM:SY5Ymock vs. UA 1µM:SY5Yapp	0.8869	0.9486 ns
